# High school graduation - the impact of older siblings' educational achievement

**DOI:** 10.23889/ijpds.v1i1.377

**Published:** 2017-04-19

**Authors:** Elizabeth Wall-Wieler, Leslie Roos

**Affiliations:** 1 University of Manitoba

## Objective

Failing to graduate high school is linked to many risk factors, including family history academic achievement. This research examines how important an older sibling's academic achievement is in predicting whether a younger sibling will graduate high school.

## Method(s)

This study used linkable administrative databases housed at the Manitoba Centre for Health Policy (MCHP). The cohort consists of 33,843 individuals born in Manitoba between April 1, 1983 and March 31, 1994, who stayed in the province until at least their 20th birthday, had at least one older sibling, and had no missing values on key variables. Logistic regression, controlling for a variety of confounders, is used to determine how much having an older sibling who didn't graduate high school impacts the odds of a younger sibling not graduating high school. 

## Results

The adjusted odds of not graduating high school within 6 years of entering grade nine for individuals who had at least one older sibling who did not graduate high school was 4.81 (p < 0.0001, 95% CI 4.4-5.2) times higher than for individuals whose older sibling(s) graduated high school. Individuals living in low income neighborhoods at birth or age 18, individuals living in rural northern Manitoba at birth or age 18, and individuals who moved before age 18 were significantly less likely to finish high school. High school graduation rates for those living in the lowest income quintile at age 18 whose older siblings graduated high school were higher than those living in the highest income quintile at age 18 and had at least one older sibling who did not graduate high school.

## Conclusion

The influence of an older sibling's educational achievement has significant implications for younger siblings' odds of high school graduation. This is likely due to social learning (younger sibling modeling actions of older sibling), and the shared parental influence and social risk experienced by both siblings.

